# A cluster-randomized educational intervention to reduce inappropriate prescription patterns for elderly patients in general practice – The Prescription Peer Academic Detailing (Rx-PAD) study [NCT00281450]

**DOI:** 10.1186/1472-6963-6-72

**Published:** 2006-06-11

**Authors:** Jørund Straand, Arne Fetveit, Sture Rognstad, Svein Gjelstad, Mette Brekke, Ingvild Dalen

**Affiliations:** 1 Department of General Practice and Community Medicine, University of Oslo, PO Box 1130 Blindern, 0317 Oslo, Norway; 2 Institute of Basic Medical Sciences, Department of Biostatistics, University of Oslo, PO Box 1122 Blindern, 0317 Oslo, Norway

## Abstract

**Background:**

Age-related alterations in metabolism and excretion of medications increase the risk of adverse drug events in the elderly. Inappropriate polypharmacy and prescription practice entails increased burdens of impaired quality of life and drug related morbidity and mortality. The main objective of this trial is to evaluate effects of a tailored educational intervention towards general practitioners (GPs) aimed at supporting the implementation of a safer drug prescribing practice for elderly patients ≥ 70 years.

**Methods/design:**

Approximately 80 peer continuing medical education (CME) groups (about 600 GPs) in southern Norway will be recruited to a cluster randomized trial. Participating groups will be randomized either to an intervention- or a control group. The control group will not receive any intervention towards prescription patterns in elderly, but will be the target of an educational intervention for prescription of antibiotics for respiratory tract infections. A multifaceted intervention has been tailored, where key components are educational outreach visits to the CME-groups, work-shops, audit and feedback. Prescription Peer Academic Detailers (Rx-PADs), who are trained GPs, will conduct the educational outreach visits. During these visits, a set of quality indicators (QIs), i.e. explicit recommendations for safer prescribing for elderly patients, will be presented and discussed. Software will be handed out for installation in participants' practice computers to enable extraction of pre-defined prescription data. These data will subsequently be linked to corresponding data from the Norwegian Prescription Database (NorPD). Individual feedback reports will be sent all participating GPs during and one year after the intervention. Feedback reports will include QI-scores on individual- and group levels, before and after the intervention. The main outcome of this trial is the change in proportions of inappropriate prescriptions (QIs) for elderly patients ≥ 70 years following intervention, compared to baseline levels.

**Discussion:**

Improvement of prescription patterns in medical practice is a challenging task. Evidence suggests that a thorough evaluation of diagnostic indications for drug treatment in the elderly and/or a reduction of potentially inappropriate drugs may impose significant clinical benefits. Our hypothesis is that an educational intervention program will be effective in improving prescribing patterns for elderly patients in GP settings.

## Background

Although appropriate use of drugs may indeed be of vital importance to elderly patients, drugs represent a double-edged sword due to their potential adverse effects. Age-related changes in drug metabolism and sensitivity put frail elderly patients at particular risk for adverse drug events (ADEs) [1,2]. The Word Health Organization (WHO) defines an ADE as a detrimental response to medication that is undesired and unintended, excluding therapeutic failure, poisoning, and intentional overdose [3]. In the elderly, between 10 and 20% of all hospital admissions are drug-related [4-8] and also associated with a higher mortality than other reasons for hospitalization [4]. In a comprehensive study from a large internal medical department in Norway, more than one out of six deaths were judged to be caused by the drug treatment used rather than the underlying illness [9]. According to the WHO, rational use of drugs implicates "each patient receiving medication appropriate for his/her clinical needs, in doses meeting the related requirements, for an adequate period of time and at the lowest cost to them and to community" [10].

Inappropriate us of drugs commonly occur in the elderly and may imply impaired quality of life and drug related morbidity and morbidity [4,11-15]. Problems related to GPs' lack of updated medication lists for their patients [16], the common practice of prescribing for patients without seeing them [17,18], inadequate communication between physicians and community nurses regarding treatment for shared care patients [16], and patients' noncompliance, as well as physicians' lacking ability to understand, detect, and improve compliance, also increase the risk of medical errors and ADEs [19].

In the Norwegian general practice *Møre & Romsdal Prescription study*, Straand and Rokstad [15] considered 13.5% of 16774 prescriptions for elderly patients to be inappropriate. Corresponding figures have been reported by Stuck et al. [20], who found that 14% of 414 patients older than 75 years used at least one inappropriate drug. Willcox et al. [17] found that potentially inappropriate drugs were prescribed for 23.5% of 6 171 patients 65 years and older. However, the different studies apply different criteria for inappropriateness. Based on Beers criteria [20], Aparasu and Mort [21] reviewed eight studies on prescription patterns in elderly, and found that the rate of inappropriate drug use varied from 14% in community-dwelling elderly to 40.3% in nursing home residents.

Prescribing indicators for general practice have been used for more than two decades [22]. Previous research has highlighted the importance of a critical approach to prescribing practice [23,24], the defining and assessment of the appropriateness of prescribing [25,26], the exploration of variations in prescribing across general practices [27], and the physicians' adherence to various standards [28]. However, few validated quality indicators exist for prescribing in general practice [29], and published standards tend to be consensus-based rather than evidence-based.

Quality indicators (QIs) are explicitly defined and measurable items which may serve as building blocks in the assessment of care [30]. As statements about structure, process, or outcomes they may be used to generate criteria and standards to measure specific aspects of clinical practice [31]. Indicators differ from guidelines, review criteria, and standards and may be regarded as a kind of rules of the thumb. Primary care rarely meets absolute standards [32], and the development of indicators must be realistic and set according to context and patient circumstances [33]. Indicators can measure the frequency with which an event occurs, such as prescriptions of a particular drug. However, indicators do not provide definite answers, they rather *indicate *potential problems. Quality measures are now increasingly being developed for primary care quality assessments and research [30]. This development has probably been encouraged by the increased access to large health care databases, made up by automated electronic records of e.g. filled prescriptions, professional services, and hospitalizations [34]. Linking prescription databases with various electronic patient record (EPR) systems comprising detailed information of patients' symptoms, findings and diagnoses enables a "reality-based" pharmaco-epidemiologic research, which may be useful for highlighting areas in need of quality improvements.

The identification of suboptimal practice is, however, only the first step for quality improvements. Several educational strategies have been used to improve doctors' clinical practice, but substantial effects are only rarely reported [35,36]. Rokstad et al. [37] performed a controlled educational intervention towards GPs aimed at improving their prescriptions for acute cystitis and insomnia. The intervention included mailed prescription feedback along with printed therapeutic recommendations, and resulted in statistical significant (although clinically moderate) improvements in prescription patterns in the intervention group (GPs in one district), compared to the control group (GPs in another district) [37]. Davies et al. [38] reviewed 777 continuous medical education (CME) studies and found evidence that educational intervention consisting of passive dissemination of clinical practice guidelines had little or no effect on practice. This corresponds with later reports by Oxman et al. [36] and Freemantle et al. [39]. More active strategies, like educational outreach visits [40,41] and multifaceted interventions [35], are more effective, but require more resources [35]. In an extensive review, Jamtvedt et al. [41] concluded that audit and feedback may be effective in improving professional medical practice, although the effects are generally moderate, and that absolute effects of audit and feedback are more likely to be larger when baseline adherence to recommended practice is low.

Underlying reasons for deviations between clinical practice and clinical guidelines vary from one problem to another and from one physician to another [42]. Therefore, it is recommended to address potential barriers to change when tailoring an intervention targeting change in medical performance. Because non-equivalent comparison groups may distort the results of evaluations, cluster-randomized trials (in which healthcare professionals or groups of professionals, rather than patients are randomized) are more likely to provide valid results than other research designs, such as controlled before-after or time-series studies [43].

We have developed an intervention aimed at improving GPs' prescribing practice for elderly (≥ 70 years) out-patients, and planned a cluster randomized controlled trial to assess the effectiveness of this intervention.

## Methods

### Design

Our hypothesis is that an educational intervention program carried out during a 6-month period will be effective in improving prescribing patterns by reducing the occurrence of potentially targeted harmful drugs and drug-drug interactions in elderly patients.

Our main hypothesis will be tested using a cluster-randomized controlled trial comparing outcomes between the intervention and control groups at follow-up. Peer continuing medial education (CME) groups of GPs will be randomized to receive a tailored intervention to support a safer prescription practice for the elderly or to a control group. The control group will be assigned to another trial, namely an educational intervention for better prescription of antibiotics for respiratory tract infections [44]. As such, the control group may also be susceptible to changes in their general prescription patterns, i.e. a possible Hawthorne effect. To control for this, we will be able to analyze prescription patterns for *all *Norwegian GPs not included in the current trial, using data from the Norwegian Prescription Database (NorPD), a national registry including data for all prescription drugs issued at Norwegian pharmacies, established in 2004 [45].

Prescription data will be collected for all eligible patients from intervention- and control group GPs at baseline and one year after the initiation of the trial. Changes in prescribing patterns will be tracked by a set of explicit recommendations (quality indicators) developed by the authors (Table 1), which will be used to analyze changes in baseline and post-intervention prescribing patterns. Degree of improvements will be measured by recording prescriptions of the particular drugs and the concurrent use of listed drug combinations that the GPs have been recommended to avoid.

### Participants

In Norway, specialists in general practice have to renew their clinical specialty every five years. In this renewal process, participation in a number of peer CME group meetings is compulsory, in order to stimulate continuously medical education and reflection. All GPs participating in such peer groups, on average consisting of six to eight peers, located in southern Norway, will be invited to participate in this trial. GPs using one of the four major EPRs (Infodoc^®^, WinMed^®^, ProfDoc Vision^® ^or System X^®^) are eligible for the trial. More than nine out of ten Norwegian GPs use one of these systems routinely during consultations with patients [46].

### Intervention

The intervention has been developed through a process of identifying potential harmful prescriptions to elderly; either as specific drugs and combinations of particular drugs to be avoided due to clinically relevant interactions, or the concurrent use of three or more psychotropic drugs. The identification of suboptimal pharmacological treatments to be targeted in this study, was based on previous research and active reflection and discussions based on own clinical experience from general practice. This process led to the development of a set of prescription QIs (Table 1).

In order to implement rational prescribing patterns, 13 GPs will be recruited as tutors, each with responsibility for the intervention in about three peer groups. Each tutor, named Prescription Peer Academic Detailer (Rx-PAD), will receive a four days' pre-study training program, focusing on safety issues in relation to pharmacological treatment in the elderly, pedagogical intervention techniques, and how to use the data-software for extracting data files from the EPRs. When recruiting the Rx-PADs, emphasis will be laid on economic independence from pharmaceutical manufacturers.

**Figure 1:**
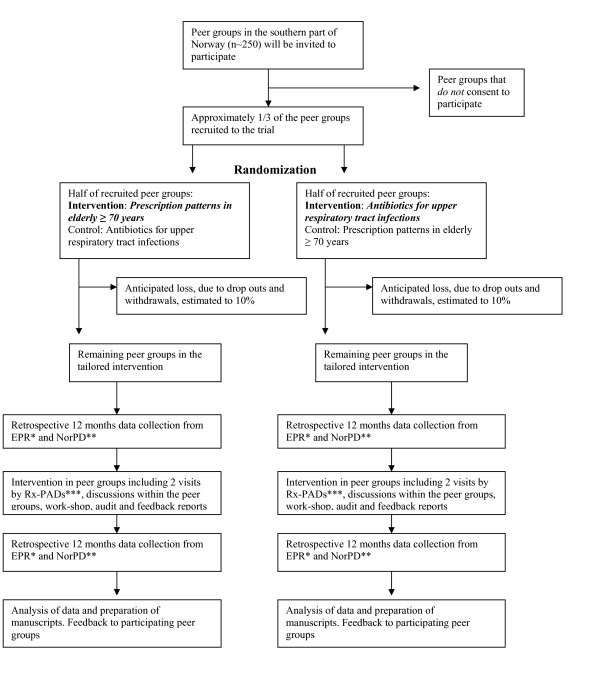
**Flow of practices through The Rx-PAD Study**. *Electronic Patient Record, **Norwegian Prescription Database, ***Prescription Peer Academic Detailer

The tailored intervention towards the GPs in the peer CME groups will include two educational outreach visits by one Rx-PAD, discussions within the peer CME group, extraction of prescription data, audit and individual feedback reports, as well as a one day work-shop with all participants, in order to facilitate reflections on personal prescription patterns. Elements in the tailored intervention are summarized in Figure 1.

In the first outreach visit, the main elements of the intervention will be presented, with special emphasis on safer prescription strategies for elderly (≥ 70 years) out-patients, choice of first-line drugs, possible harmful effects of particular drugs, and specific drug combinations to be avoided. During the visit, a software package will be delivered to the participating GPs for installation in own practice computers. This enables data extraction from the preceding 12-month period, which will comprise baseline data used in the study. Prescription data will be collected on diskettes and analyzed by research staff, followed by an individual prescription report which will be mailed to each participant.

The second outreach visit, which will take place within two months after the first one, will focus on the disclosed individual prescription patterns in relation to the explicit recommendations provided during the first meeting. The Rx-PADs will facilitate the discussion within their peer groups, based on the individual feedback reports, enabling participants to compare own prescription patterns with overall averages. This will probably trigger discussion within the peer group, aimed at critical reflection towards own prescription strategies for elderly patients and facilitating disclosure of areas where individual improvements may be desirable.

About three months after the second outreach visit, all participants will be gathered in regional work-shops where rational pharmacological treatment for elderly out-patients ≥ 70 years will be outlined in more depth, on the basis of the baseline prescription data.

Twelve months after the first data extraction, a second data extraction addressing the preceding 12-months prescription patterns, will be made. Each GP will then get a new feedback report revealing before- and after-intervention-data in relation to the QIs and compared with average figures for the total sample. Application will be sent to The Norwegian Medical Association (NMA) to have this educational intervention approved as a so called "clinical theme-course", which will give the participants important CME credit.

### Quality indicators (QIs)

In this study we have developed 14 explicit QIs regarding possible inappropriate prescription patterns to elderly out-patients ≥ 70 years, based on current knowledge and pervious research [15,47-52] (Table 1). The face validity of the QIs will be assessed using a modified three postal round Delphi consensus technique [47,53,54]. The Delphi technique is a structured interactive method involving repetitive administration of anonymous questionnaires, usually across two or three postal rounds [30]. There is limited evidence of the content validity of quality measures derived using the Delphi technique [53,55]. Still, the Delphi procedure has been used for validating the relevance of prescribing indicators [29], i.e. concerning GP's prescribing of non-steroidal anti-inflammatory drugs [47], as well as indicators for patient and general practitioner perspectives of chronic illness [23], and indicators for cardiovascular disease [56]. We will recruit a panel of health professionals (GPs, clinical pharmacologists and geriatricians; about 20 in each group) to rate the face validity of our proposed QIs. Also, the participating GPs will be invited to assess their interpretation of the QIs used in this study by the end of the six-month educational intervention. This will be made by filling in a questionnaire where the relevance of each QI may be scored on a visual-analogue scale.

### Data handling

Software aimed at extracting predefined data sets will be developed for this trial. The software will be compatible with the four electronic patient record (EPR) systems (Infodoc^®^, WinMed^®^, ProfDoc Vision^® ^or System X^®^) used by the waste majority of Norwegian GPs. In addition we will provide technical telephone support during the whole data collection period. The participants will get a deadline for sending their data sets, before they are able to receive an individual feedback report, and the Rx-PAD will remind their group to send data sets before the second peer group meeting.

Data extraction will provide information on contacts with patients and prescriptions issued. Prescription data will be described in terms of The Anatomical, Therapeutic, Chemical (ATC) classification system with Defined Daily Doses (DDDs), in short: the ATC/DDD system [57].

In order to merge prescription data provided by NorPD with data from the EPR systems, the Civil Personal Registration (CPR) number for each patient will be extracted from the EPR. These are the unique identification numbers for Norwegian citizens, and will be deleted from the research database after the record linkage has been performed. The dataset from NorPD will provide data on drugs actually dispensed.

**Figure 2:**
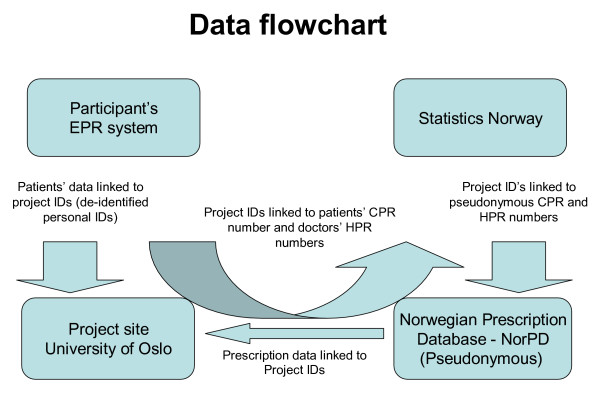
**Logistics of data collection**. Flow-chart of merging process of prescription data provided by the Norwegian Prescription Database (NorPD) and data from the electronic patient record (EPR) systems. Patients' Civil Personal Registration (CPR) numbers are unique Norwegian residents' identification keys. Health Personnel Registration (HPR) numbers are unique Norwegian health personnel registration keys. Identifiable data will be deleted from the research database when the merge is completed, as the de-identified personal IDs will be sufficient for the subsequent data analysis. Statistics Norway, which is the public institution in Norway responsible for collecting, analyzing and disseminating official statistics, will provide CPR and HPR pseudonyms making it possible to merge the two data sources.

Extracted data from the EPR will be encrypted, stored on a diskette and mailed to the principal researcher. A flow-chart of the data collection is summarized in Figure 2.

### Outcome measures

Primary outcome measures will be changes in prescription patterns after the tailored educational intervention regarding the listed QIs (Table 1). Post-intervention prescription data will be collected one year after the collection of the baseline prescription data. Prescription patterns in the intervention group will be compared with the non-intervention control group, and with corresponding data (from NorPD) based on a random sample of Norwegian GPs not included in the current trial.

In addition to investigating changes in each QI, we will summarize changes for all QIs (Table 1), in order to give a total sum score for improvement potentials. The difference in total QI sum score between the intervention group and the control group will be a main outcome measure. The QI regarding regular use of the EPR function showing patient's total regular medications will not be included in this sum score. Outcome measures are summarized in Table 2.

It would also be interesting to investigate if this intervention may influence all-over mortality patterns for elderly patients targeted during this intervention as compared to corresponding elderly patients in the control group [44]. By 2006, death data are not yet included in the NorPD, but efforts are currently undertaken to include such data in the NorPD on a regular basis. A possible mortality study has not yet been elaborated in details, but this will be considered.

### Recruitment and randomization

Block-randomization of peer groups will be done within geographical strata, each comprised of one or more counties. The size of the blocks will vary according to the number of recruited groups within each stratum. An independent researcher not involved in the study will be responsible for the randomization. Based on this randomization, peer CME groups will be assigned to either the intervention group or the control group.

### Pilot study

With the exception of the one-day CME-course, the intervention will be piloted in one CME group before the main trial.

### Ethics and data security

Physicians participating in the peer CME groups will be given information about the objectives of the study and the practical implications this may have on their practice. They will be told that each peer CME group will be randomized to one out of two interventions (improved prescription patterns towards elderly ≥ 70 years, or improved antibiotic prescriptions for respiratory tract infections), and that they will serve as control-group for the intervention they do not take part in. Participation and data extraction are based on written, informed consent from all physicians. The aim of this study is improved quality according to well established principles for good clinical practice and should therefore not imply risks for the patients involved, and we find it unlikely that the current intervention might worsen the quality of care. The project has been presented for The Regional Committee for Research Ethics and approval from The Norwegian Social Science Data Services (NSD) has been obtained, implicating acceptance to extract prescription data. We have assessed the risk for misusing the collected data, and found this unlikely due the fact that the data are linked to de-identified ID numbers. In order to use patient identification data in the record linkage of the NorPD and EPR databases, The Norwegian Directorate for Health and Social Affairs have approved a dispensation from the Health-Professional Secrecy regulations.

### Sample size and statistics

In a pilot study (including 13 physicians) for the present study, the mean number of positive hits on our listed QIs (Table 1) made up 25 hits per 100 elderly patients during the preceding 12 months period. In accordance with this finding, we anticipate an occurrence of inappropriate prescription of about 25 hits per 100 elderly patients in the present study.

Proving a clinical relevant reduction from 25 to 17 hits or less per 100 patients (32% relative reduction), assuming that the prevalence of inappropriate prescriptions in the control group remains constant, will imply a need for 74 peer groups in total (power of 80%; statistical significance level of 5%). This estimation takes into account the need to adjust for intra-cluster correlation, since data within the peer CME groups cannot be assumed to be independent. Furthermore, we assume the average number of GPs per peer CME group to be 7 (allowing for some withdrawals) and that the average number of patients ≥ 70 years per participating GP during the study period will be 165. Based on previous studies [46,58], the intracluster correlation coefficient is assumed to be 0.085, resulting in a variance inflation factor of 99 [59].

We will carry out post-test comparisons between the intervention and control group using cluster-adjusted chi-square and *t*-tests. Analysis of covariance may be used to adjust for imbalance of baseline levels between the intervention- and control-groups [60]. The data set will primarily be analyzed according to the cluster randomization, however, analyzes at physician- and sub-groups of physicians as well as compared to NorPD-data for GPs not participating in the study, will also be performed.

## Competing interests

SG has interests in Mediata Ltd, which has developed the software for the project. The other authors declare that they have no competing interests.

## Authors' contributions

JS had the original idea for the study and made the first draft of the study protocol (in Norwegian). AF prepared the current manuscript in cooperation with the authors. All authors have participated in the planning of the study and have approved the final version of this manuscript.

## Pre-publication history

The pre-publication history for this paper can be accessed here:



## Figures and Tables

**Table 1:** Quality indicators (QIs) for optimal prescription patterns for elderly ≥ 70 years

	**Quality Indicator**	**Aim**	**Concern**
**1.**	Use of an explicit list of regular medications when prescribing from the GP's electronic medical record.	The share (% of GPs using an explicit list of regular medication as default list) should be as high as possible.	Suggests that GPs have a more complete view of their patients' total regular medications.
**2.**	Tricyclic antidepressants: • Amitryptiline • Doxepin • Trimipramine	The share (% of individuals ≥70 years) should be as low as possible.	Strong anticholinergic and sedative properties increase the risk for impaired cognitive functioning in the elderly. Increased risk for urinary retention (males), constipation, impaired vision, and falls and fractures.
**3.**	1st. generation antihistamines: • Dexchlorpheniramine • Promethazine • Alimemazine • Hydroxycin	The share (% of individuals ≥ 70 years) should be as low as possible.	Strong anticholininergic properties. Should be avoided in the elderly. Especially Promethazine and Alimemazine may cause extrapyramidal ADEs.
**4.**	1st. generation (low-potency) antipsychotics: • Chlorpromazine • Chlorprotixene • Levoprometazine • Prochlorperazine	The share (% of individuals ≥ 70 years) should be as low as possible.	Strong anticholinergic and sedative properties. May cause extrapyramidal and orthostatic ADEs. Prochlorperazine has no documented effect on gait problems in the elderly.
**5.**	Long acting benzodiazepines: • Nitrazepam • Flunitrazepam	The share (% of individuals ≥ 70 years) should be as low as possible.	Long half-life and risk of accumulation may produce prolonged sedation, and cause falls and fractures.
**6.**	The muscle relaxant Carisoprodol.	The share (% of individuals ≥ 70 years) should be as low as possible	Poorly tolerated in elderly patients. Anticholinergic properties. Sedative and relaxing properties, with increased risk of falls and fractures.
**7.**	Strong analgesics poorly tolerated by the elderly: • Propoxyphene • Pethidine • Opioids with spasmolytics	The share (% of individuals ≥ 70 years) should be as low as possible.	Propoxyphene is poorly tolerated by the elderly. Pethidine may cause convulsions and renal failure. Ketobemidone/diphelyldimetylaminobutene has anticholinergic properties.
**8.**	Long term oral use of Theofylline	The share (% of individuals ≥ 70 years) should be as low as possible.	Narrow therapeutic index. Poorly documented effect on Chronic Obstructive Pulmonary Disease (COPD).
**9.**	Combination of a systemic beta blocking agent with an unselective calcium channel blocker: • Verapamil, or: • Diltiazem	The share (% of individuals ≥ 70 years) should be as low as possible.	A nonselective calcium channel blocker in combination with a beta blocking agent may cause myocardial depression and atrioventricular heart block.
**10.**	Combination of NSAID (non-steroid anti-inflammatory drug) and Warfarin.	The share (% of individuals ≥ 70 years) should be as low as possible.	Increased risk for gastrointestinal bleedings with or without elevated INR-levels.
**11.**	Combination of NSAID (or a Cox2-inhibitor) and ACE-inhibitor (or an A2-blocker).	The share (% of individuals ≥ 70 years) should be as low as possible.	May cause kidney failure in elderly patients, particularly if presence of general arteriosclerosis, dehydration or concurrent use of diuretics.
**12.**	Combination of NSAID and SSRI (selective serotonin reuptake inhibitors).	The share (% of individuals ≥ 70 years) should be as low as possible.	The combination of NSAID and SSRI increases the risk of gastrointestinal bleeding.
**13.**	Combination of NSAID and diuretics.	The share (% of individuals ≥ 70 years) should be as low as possible.	May reduce the effect of diuretics and worsen existing heart failure.
**14.**	3 or more psychotropic drugs: • N02A* Analgesics containing opioids • N05* Psycholeptics: 1. antipsychotics 2. hypnotics 3. tranquillizers • N06A* Antidepressants	The share (% of individuals ≥ 70 years) receiving three or more different psychotropic drugs should be as low as possible.	Increased risk for excessive sedation, interactions and central nervous adverse effects. Increased risk for falls and fractures.

* Anatomical Therapeutic Chemical (ATC) classification code.

**Table 2:** Outcome measures in study of the effect of a pedagogic intervention towards groups of Norwegian GPs, concerning inappropriate prescription patterns in elderly patients ≥ 70 years

Baseline prescription patterns collected during one year:
• Proportion of inappropriate prescriptions for elderly patients ≥ 70 years

Change (%) in prescription patterns compared to baseline in elderly patients ≥ 70 years, regarding the following drugs and combination of drugs, one year after the initiation of a tailored pedagogic intervention:
• Tricyclic antidepressants (Amitriptyline, Doxepin, Trimipramine)
• 1^st ^generation antihistamines (Dexchlorphenamine, Promethazine, Alimemazine, Hydroxycin)
• 1^st ^generation low potency antipsychotics (Clorpromazine, Chlorprotixene, Levoprometazine, Prochlorperazine)
• Long acting benzodiazepines (Nitrazepam, Flunitrazepam)
• The muscle relaxant Carisoprodol
• Strong analgesics poorly tolerated by the elderly (Propoxyphene, Pethidine, opioids with spasmolytics)
• Theophylline per os
• Beta blocking agents combined with unselective calcium channel blockers (Verapamil, Diltiazem)
• NSAIDs combined with Warfarin
• NSAIDs or Cox2-inhibitor combined with ACE-inhibitor or A2-blocker
• NSAIDs combined with SSRI
• The difference in "total prescription change score" between the intervention group and the control group

Prescription logistic issues:
• Agreement between drugs listed as "regular" in the physicians' electronic medical record (EPR) systems and dispensed drugs, registered in the Norwegian Prescription Database (NorPD)
• Agreement between prescribed and dispensed drugs, registered in the Norwegian Prescription Database (NorPD)

Methodological issues:
• Development of rational prescription patterns and related quality indicators (QIs) for elderly out-patients ≥ 70 years
• Feasibility evaluation of a large cluster randomized educational intervention study in general practice settings
